# A new device for simple and accurate urinary pH testing by the Stone-former patient

**DOI:** 10.1186/2193-1801-3-209

**Published:** 2014-04-28

**Authors:** Felix Grases, Adrian Rodriguez, Francisco Berga, Antonia Costa-Bauza, Rafael Maria Prieto, Isabel Burdallo, Alfredo Cadarso, Cecilia Jimenez-Jorquera, Antonio Baldi, Rosendo Garganta

**Affiliations:** Laboratory of Renal Lithiasis Research, Faculty of Sciences, University Institute of Health Sciences Research (IUNICS-IdISPa), University of Balearic Islands, Ctra. de Valldemossa, km 7.5, 07122 Palma de Mallorca, Spain; Instituto de Microelectrónica de Barcelona, IMB-CNM (CSIC), Campus UAB, E-08193 Cerdanyola, Spain; Devicare, Av. Diagonal 327, C-2., E-08009 Barcelona, Spain

**Keywords:** Urinary pH, Renal lithiasis, pH measurement, pH self-control

## Abstract

**Purpose:**

Urinary pH is an important factor linked to renal stone disease and a useful marker in the treatment of urolithiasis. Although the gold standard for measuring urinary pH utilizes a glass electrode and a pH meter, at present dipstick testing is largely used to estimate urinary pH. However, the accuracy and precision of this method may be insufficient for making clinical decisions in patients with lithiasis. The aim of this study is to describe a new device for urinary pH testing.

**Methods:**

The device includes a pH sensor based on differential measurement of an ISFET-REFET pair. The drawbacks associated with this type of configuration, namely short lifetime and manual fabrication, have been overcome in the prototype. An automatic one point calibration is performed when turning on the system. Two buffer solutions were utilized to determine the intra- and inter-day precision of the device. The pH of 30 fresh human urine samples was measured using a pH-meter, a dipstick and the new electronic device.

**Results:**

In some cases, dipstick measurements differed from those of the pH meter by more than 0.40 units, a clinically relevant discrepancy, whereas none of the measurements made with the new electronic device differed from the results of the pH-meter by more than 0.1 pH units.

**Conclusions:**

This new electronic device has the possibility to be used by stone-formers to control their urinary pH at home, increasing the tools available for stone prevention and prophylaxis.

## Introduction

Urinary pH is an important factor linked to renal stone disease (Hess 2006). Although the formation of calcium oxalate crystals (either mono- or dihydrate) is apparently unrelated to urinary pH, because the solubilities of these salts are practically unaltered at urinary pH values, a urinary pH <5.5 may induce the formation of uric acid crystals whereas a urinary pH >6.2 may induce the formation of calcium phosphate crystals.

The formation of calcium oxalate crystals in urine takes place through heterogeneous nucleation processes (Grases et al. 2012; Finlayson 1978). For this reason, the presence of solid preformed particles is an indispensable condition for the formation of any type of calcium oxalate stones.

Calcium oxalate monohydrate (COM) papillary calculi initiate their formation in injured papillary tissue, mainly through calcification of this tissue by hydroxyapatite. When these deposits grow and erode the epithelium covering the papillae, they come into contact with urine. Due to the permanent supersaturation of calcium oxalate in urine, and through heterogeneous nucleation, COM papillary calculi start to develop (Grases et al. 2013).

For this reason, COM papillary calculi are unique among renal calculi, since their formation is independent of urinary pH (Grases et al. 2012). For the remainder calcium oxalate stones, the presence of an adequate number of solid preformed microparticles in urine is an indispensable condition for their formation.

At urinary pH < 5.5, uric acid becomes insoluble and forms crystals of anhydrous or dihydrate uric acid (Grases et al. 2000). If the amounts of uric acid are considerable, uric acid or mixed uric acid/COM stones will form. As expected, these types of stones were more prevalent in patients with urinary pH < 5.5 than in those with higher urinary pH (Liebman et al. 2007). If the amount of formed uric acid crystals is small, it can be eliminated as asymptomatic crystalluria. However, due to the ability of anhydrous uric acid crystals to act as heterogeneous nucleant of COM, the development of these types of calculi may be favored under appropriate conditions of calcium oxalate supersaturation and a deficiency of crystallization inhibitors (Grases et al. 2002,2007). The capacity of uric acid to serve as heterogeneous nucleant of COM crystals is superior to that of mucin (a glycoprotein) or cellular detritus, but inferior to that of some calcium salts (Grases et al. 2007). Moreover, uric acid was detected as a minor component in the core of unattached COM renal calculi, indicating that the core was principally formed by COM crystals, uric acid and organic matter (Grases et al. 2006). In such calculi, uric acid probably played an important role as an heterogeneous nucleant of COM crystals. Thus, uric acid was responsible for calculus formation. Hence, it is not surprising that a majority of COM unattached renal stones (50,9%) developed in patients with urinary pH < 5.5 (Grases et al. 2012).

At urinary pH > 6.2, calcium phosphates (hydroxyapatite and brushite) form insoluble salts (Söhnel & Grases 2011). If large amounts are present, hydroxyapatite or brushite stones will develop (Grases et al. 2012). If smaller amounts of these crystals are present, they cannot form these types of stones. However, due to their capacity to induce calcium oxalate dihydrate heterogeneous nucleation (Wu et al. 1999), they may favor the formation of corresponding calculi. In fact, it was found that calcium oxalate dihydrate calculi (41,5%) were mainly present in patients with urinary pH > 6.0 (Grases et al. 2012).

All the commented aspects demonstrate the importance of precise urinary pH measurements. Due to its accuracy and precision, the gold standard for pH measurements utilizes a glass pH electrode and a pH meter. At present, however, dipstick testing is largely used to estimate urinary pH. Use of a pH meter is more cumbersome, due to the requirement for regular calibration and the need for user training. In contrast, dipsticks are cheaper and do not require user training or calibration with test solutions. Thus, patients can use dipsticks to measure their own urinary pH at home, avoiding the drawbacks caused by not testing freshly voided samples. However, accuracy and precision of dipstick pH measurements are insufficient for making clinical decisions on patients with lithiasis (Kwong et al. 2013; Desai and Assimos 2008).

This paper describes a new, easy to use device for measuring urinary pH, suitable for use by lithiasic patients at home. This new device is based on a silicon sensor (Ion Selective Field Effect Transistor, ISFET), with several advantages compared with the combination of a pH meter and glass electrode: 1) greater mechanical robustness; 2) no need for maintenance; and 3) fabrication and packaging with Integrated Circuit technologies, enabling mass production at extremely low cost.

## Materials and methods

### Subjects and samples

The urine of 30 human volunteers was collected into sterile receptacles. Each sample was immediately tested twice by two fully trained operators using three methods: a calibrated pH-meter (Crison S.L. Barcelona, Spain), urine dipsticks (Merck, Darmstadt, Germany) and the new electronic device described below (Devicare, Barcelona, Spain). Samples were tested in random order.

Each volunteer provided written informed consent, and the study protocol was approved by the institutional review board of the Balearic Islands Community (no. IB 2029/13 PI).

To establish the accuracy of each method, and to evaluate the intra- and inter-day precision of the new device, two buffer solutions were used (0.7 M acetate buffer, pH 5.5; and 0.15 M phosphate buffer, pH 6.5). The pH of each buffer was measured five times using each device on each of two days.

### Description and characteristics of the new electronic device for urinary pH testing

**Figure 1:**
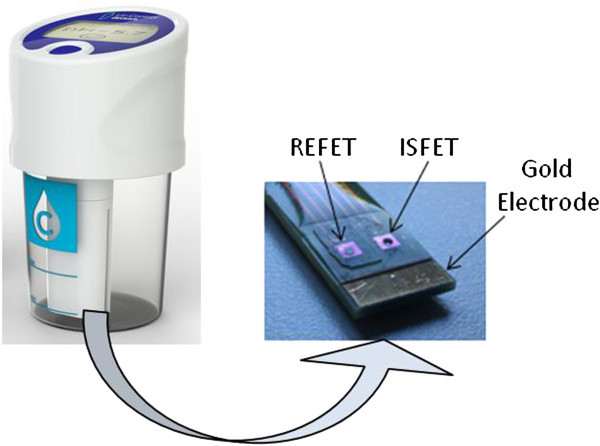
**Images of the device (left), and pH sensor probe (right).**

**Figure 2:**
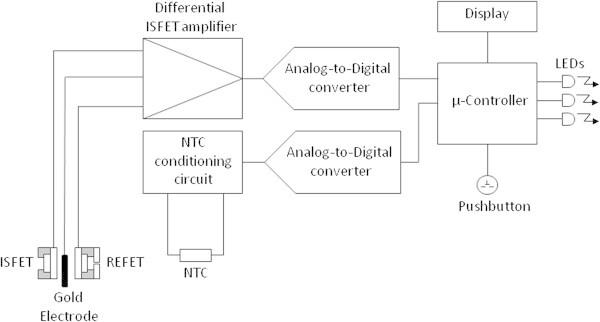
**Scheme of the experimental set-up.**

The device resembles a standard urine specimen cup with a special lid (Figure 1). This lid includes a pH sensor probe that protrudes into the liquid contained in the cup and electronic circuits for the sensor signal readout and pH calculation. The scheme of the prototype is shown in Figure 2. The external top surface of the lid includes a pushbutton and a display with the measured pH. The device is also provided with a cup containing pH 6.00 buffer. When the device is not being used, the lid is left screwed onto the buffer cup. The device is activated by pushing the button, automatically triggering a one point calibration. The lid is removed from the calibration cup and placed onto the specimen cup, which has been previously filled with the sample to be tested. The button is pushed again to measure the pH within a measure time inferior to 10 sec. The measured pH then appears on the display.

The pH sensor is based on the differential measurement of two Ion Selective Field Effect Transistors (ISFETs), one modified to work as a reference ISFET (REFET) (Compte and Janata 1978). The REFET is simply an ISFET with a micro-chamber containing buffer solution wetting its sensitive gate. The micro-chamber is connected to the external solution through a microchannel liquid junction. The drawbacks associated with this type of configuration, namely short lifetime and manual fabrication (Guth et al. 2009), have been overcome in the prototype described here. By keeping the sensor in the buffer solution between measurements, the solution in the REFET micro-chamber is continuously changed by diffusion. The dimensions of the microchamber and microchannel are designed to withstand more than 24 h measurements without significant contamination of the internal buffer solution. In the present prototype, the REFET microchamber and microchannel were fabricated from diacrylate bisphenol, a photocurable polymer used for packaging, and subsequent bonding of a micromolded PDMS lid (Burdallo et al. 2012).

## Results

The intra- and inter-day precision of the new device was tested using two buffer solutions. The standard deviation obtained for both showed excellent results (Table 1).

**Table 1:** **Mean and standard deviation of pH values of two buffer solutions measured with the new electronic device**

	0.7 M acetate buffer pH = 5.5		0.15 M phosphate buffer pH = 6.5	
	Mean	Standard deviation	Mean	Standard deviation
Intra-day (n = 5)	5.42	0.04	6.50	0.00
Inter-day (n = 5)	5.46	0.05	6.50	0.00

**Figure 3:**
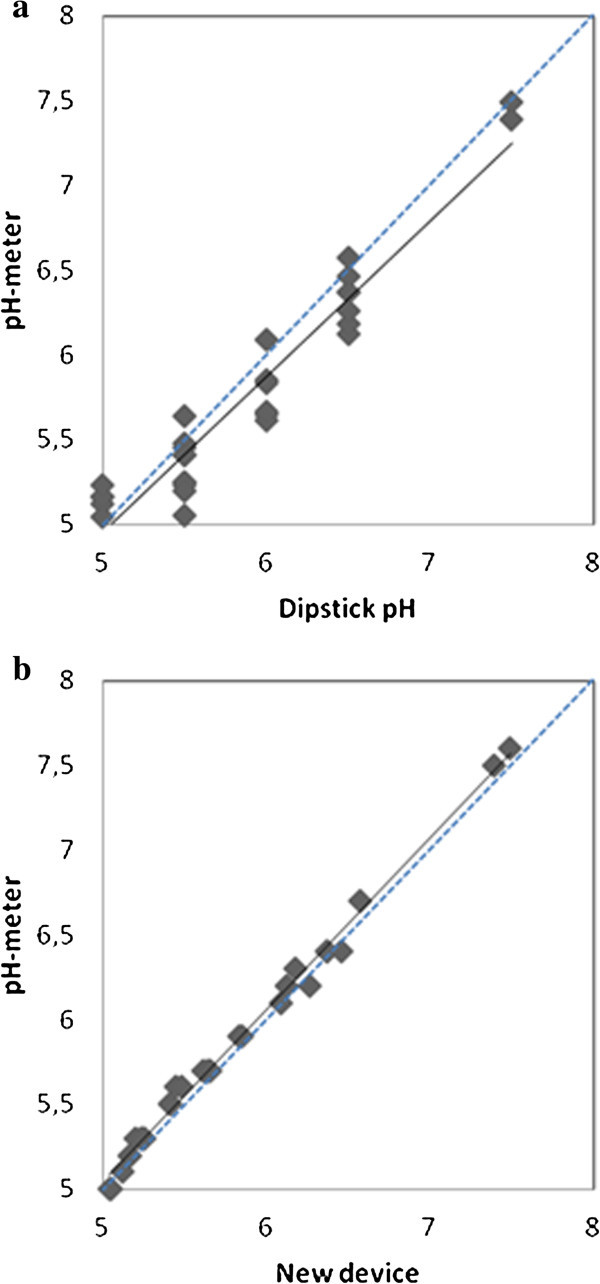
**Scatter graphs showing the correlation of pH measurements between pH meter and the dipstick (3a) and the new device (3b) in fresh urine.** The regression line is illustrated by a solid line, while the unity line is shown in dotted.

The pH of 30 fresh urine samples was measured using three methods: a pH-meter, a dipstick and the new electronic device (Figure 3). The Pearson correlation coefficients for the dipstick and the new device were 0.922 and 0.993, respectively.

A regression of results obtained with the pH-meter vs results obtained with the new device led to a linear graph comparable to the theoretical line with slope = 1 and intercept = 0 at the 95% confidence level (slope = 0.982 ± 0.019; intercept = 0.06 ± 0.11).

Table 2 summarizes the pH of fresh urine samples obt ained with the dipstick and the pH meter in fresh urine. The urinary pH measured with the dipstick was not sufficiently accurate in patients diagnosed with lithiasis, with differences between the two devices as high as 0.4 pH units. In contrast, the new electronic device was more accurate and precise in measuring pH in fresh urine (Table 3), with a maximum error of 0.1 pH units.

**Table 2:** **pH values measured with the dipstick and the pH-meter in fresh urine**

pH dipstick	pH-meter Mean (±SD)	Difference (±SD)	n
5,0	5,14 ± 0.07	0,14 ± 0.07	5
5,5	5,34 ± 0.17	0,16 ± 0.17	10
6,0	5,78 ± 0.18	0,22 ± 0.18	6
6,5	6,33 ± 0.16	0,17 ± 0.16	7
7,0			0
7,5	7,44 ± 0.07	0,06 ± 0.07	2

**Table 3:** **pH values measured with the new electronic device and the pH-meter in fresh urine**

pH interval	pH-meter Mean (±SD)	New device Mean (±SD)	Difference (±SD)	n
5-5.5	5,17 ± 0.08	5,20 ± 0.12	0.03 ± 0.14	10
5.5-6	5,60 ± 0.14	5,68 ± 0.14	0.08 ± 0.20	10
6-6.5	6,26 ± 0.14	6,29 ± 0.12	0.03 ± 0.18	7
6.5-7.5	7,17 ± 0.50	7,27 ± 0.49	0.10 ± 0.70	3

The characteristics of renal lithiasis indicate that the maximum allowable error for urinary pH is 0.1 pH units. None of the pH measurements made using the new electronic device differed from those made by the pH meter by more than 0.1 pH units. However, more than 70% of the differences between the dipstick and the pH meter were above 0.1 pH units, and more than 40% had an absolute difference of more than 0.2 pH units.

## Discussion

The present study confirmed previous results (Kwong et al. 2013) demonstrating that dipstick and pH meter measurements of urinary pH differ markedly, with some of these differences being clinically relevant. In the present work, the pH of some urine samples differed by up to 0.40 pH units, suggesting that the dipstick method may be unreliable in making clinical decisions (Desai and Assimos 2008).

It is important for uric acid stone-formers and those with calcium oxalate calculi induced by uric acid crystals to distinguish between urinary pH above and below pH 5.5. Moreover, non-infected calcium phosphate stone-formers and patients with calcium oxalate stones induced by calcium phosphate crystals must distinguish between urinary pH above and below pH 6.2. The present findings suggest, however, that the dipstick method may be unable to accurately distinguish between urinary pH above and below these cutoff points. These errors can result in unnecessary (or excessive) urine alkalinization or acidification during the implementation of prophylactic treatments. In contrast, the new electronic device described here showed good intra-day and inter-day precision and small (<0.1 pH unit) absolute differences from a pH meter when measuring the pH of fresh urine samples. Taken together, these results demonstrate that this new electronic device is suitable for the evaluation of urinary pH in stone-formers.

The proposed electronic device has an autocalibration system, allowing urinary pH to be measured immediately after pushing the on button. Moreover, the design of the device suggests that it can be easily operated by an untrained user, such as a patient at home. Finally, to this device it could be easily introduced the automated storage and processing of recorded pH values. Patients can therefore readily and more immediately control their urinary pH by modifying their alkalinizing or acidifying therapy, as well as avoiding errors resulting from urine transport and storage.

In conclusion, this new electronic device has the possibility for use by stone-formers in precise urinary pH control at home, increasing the tools available for stone prevention and prophylactic control.

## Ethical standards

The study protocol was approved by the ethical standards of the Balearic Islands Community (no. IB 2029/13 PI) and has been performed in accordance with the ethical standards laid down in the 1964 Declaration of Helsinki and its later amendments.

All volunteers provided their written informed consent prior to their inclusion in the study.
